# SNARE-ing the Reason for Post-Cardiac Surgery Critical Illness-Related Corticosteroid Insufficiency

**DOI:** 10.3390/genes15010128

**Published:** 2024-01-20

**Authors:** Nicholas Diehl, Natalia Kibiryeva, Jennifer Marshall, Sarah L. Tsai, Juan S. Farias, Jaime Silva-Gburek, Lori A. Erickson

**Affiliations:** 1 Graduate Medical Education, Kansas City University, Kansas City, MO 64106, USA; 2 Biosciences, Kansas City University, Kansas City, MO 64106, USA; nkibiryeva@kansascity.edu; 3 Ward Family Heart Center, Children’s Mercy Kansas City, Kansas City, MO 64108, USA; 4 Strategy, Innovation, and Partnerships, Children’s Mercy Kansas City, Kansas City, MO 64108, USA; jamarshall@cmh.edu; 5 Endocrinology, Children’s Mercy Kansas City, Kansas City, MO 64108, USA; sltsai@cmh.edu; 6 School of Medicine, University of Missouri-Kansas City, Kansas City, MO 64108, USA; jcsilvagburek@cmh.edu; 7 Graduate Medical Education, Children’s Mercy Kansas City, Kansas City, MO 64108, USA; jsfariastorres@cmh.edu; 8 Department of Critical Care, Children’s Mercy Kansas City, Kansas City, MO 64108, USA

**Keywords:** adrenal insufficiency, congenital heart disease, critical illness-related corticosteroid insufficiency, CIRCI, STX1A, genetic evaluation

## Abstract

Critical illness-related corticosteroid insufficiency (CIRCI) can cause hemodynamic instability in neonates after congenital heart surgery with manifestations that increase morbidity and potential mortality. We retrospectively reviewed neonates who underwent cardiac surgery between August 2018 and July 2020 at a freestanding children’s hospital, had next-generation sequencing performed, and had their cortisol levels drawn as standard clinical care after cardiac surgery. The groups were defined as CIRCI (with a cortisol level ≤ 4.5 mcg/dL) and non-CIRCI (level > 4.5 mcg/dL). The CIRCI group (n = 8) had a 100% incidence of heterozygous gene mutation on STX1A with splicing or loss of function, and this mutation was not found in the non-CIRCI group (n = 8). Additional gene mutations were found in the CIRCI group on RAB6A, ABCA3, SIDT2, and LILRB3, with no incidence in the non-CIRCI group. Three additional mutations were found across the CIRCI group in INPPL1 and FAM189A2 (both splicing and missense), with 12–25% of patients in the non-CIRCI group also displaying these mutations. Novel genetic abnormalities were seen in neonates with symptoms of CIRCI with potential cardiac implications from a gene mutation for STX1A. Compounding effects of additional gene mutations need to be confirmed and explored for potential predisposition to hemodynamic instability during times of stress.

## 1. Introduction

Children undergoing cardiac surgery may receive corticosteroids pre-operatively to temper cardiopulmonary bypass-related inflammation, post-operatively for hemodynamic instability, and peri-extubation to reduce airway edema. This practice has become standard in many institutions, with children typically receiving corticosteroids pre- and post-operatively to limit the detrimental effects of the inflammatory response. While most neonates undergoing congenital heart disease (CHD) surgery have an appropriate adrenocortical stress response post procedure with these perioperative precautions, abnormal cortisol levels and relative adrenal insufficiency post-operatively can manifest in the form of significant hemodynamic instability in a subset of these infants (4%) [1]. The treatments for significant hemodynamic instability include fluid boluses, high levels of inotropic support, prolonged intubation needs, extracorporeal membrane oxygenation support (ECMO), and high-dose stress steroid replacement [2,3]. Secondary stress-related adrenal insufficiency causing cardiovascular collapse is a rare but potential cause of mortality in children, with varying rates of 2–25% [1,3,4]. Still, the clinical, biochemical, and genetic-specific etiologies vary, and the actual cause of death may be multifactorial [4]. Critical illness-related corticosteroid insufficiency (CIRCI) can cause significant hemodynamic instability in neonates after congenital heart surgery with manifestations that increase morbidity and potential mortality by up to 17% [1,3]. 

Adrenal insufficiency is generally defined as a post-operative random cortisol level of less than 4.5 mcg/dL without stimulation testing [5,6]. Recent research has noted several specific genes and proteins potentially related to relative adrenal insufficiency [4,7]. The dysregulation of FKBP5 is associated with the development of several varying pathological phenotypes, which may be necessary in glucocorticoid signaling, acting as a co-chaperone for numerous steroid receptors [7]. Despite multiple studies conducted in pediatric cardiology, including randomized control trials, the cause of the variable rate of significant CIRCI response to post-operative stress and outcomes is still unknown [3,5,6]. There is also scientific debate as to whether corticosteroids are necessary in all pediatric cardiac surgery patients [8,9]. This debate allows for the postulation that administering steroids only to patients with a higher propensity to CIRCI could enable faster post-operative recovery by means of a more tailored therapeutic method. 

Very little work has been carried out specifically with pediatric cardiology patients to identify which children would benefit from exogenous steroids via a personalized medicine approach. No specific gene markers are currently used to screen children who would benefit from this evaluation prior to cardiac surgery [4]. As human biomarker evaluations are increasingly able to be rapidly completed, especially for specific genes, we sought to identify possible genetic differences in neonates with post-cardiac surgery CIRCI. 

## 2. Materials and Methods

Subjects were given a study ID as the only linking number to connect the data analyses to a specific study subject. This linking number was not included in the genetic data. The neonates were then grouped by their post-operative cortisol levels: ≤4.5 mcg/dL for the CIRCI group and >4.5 mcg/dL for the non-CIRCI group. All genetic analyses were separated into the cohorts of a non-CIRCI post-operative cortisol level group versus the CIRCI neonates, and de-identified genetic analyses were performed based on these two groupings. 

This study consisted of a single site, de-identified, retrospective, descriptive cross-section of term neonates who underwent pediatric CHD surgery from August 2018 to July 2020. The inclusion criteria additionally involved post-operative cortisol level measurements and next-generation DNA sequencing performed as per the standard clinical care through our institution’s genome center. A retrospective chart review was completed for the demographics and clinical course of the variables listed in Table 1. The demographics of the sample were compared with descriptive findings of incidence, ranges, mean, and medians and analyzed with SPSS. Due to the small sample size and non-normal distribution, nonparametric tests were used including the Mann–Whitney U, Fisher’s exact, and Kruskal–Wallis tests, respectively, for continuous, ordinal, and multiple categorical variable types. 

The genomic variants were studied using next-generation sequencing (NGS). Firstly, FASTQ files generated with Illumina and provided by the Core Genome facility at Children’s Mercy Hospital were analyzed using PartekFlow build 11.0.24.0102 (Partek Inc, St. Louis, MO, USA) software. We used the Bowtie2 aligner algorithm, the hg38 reference genome, and the Partek Genotype likelihood algorithm to identify a list of aberrations for each subject, producing variant call files (vcf.). The resulting vcf. files were transferred to the QIAGEN Clinical Insight Interpreter (QCI) (Qiagen, Redwood City, CA, USA) database to evaluate the clinical relevance of the coding variants in the eight CIRCI patients included in the study compared to some control subjects. For our analysis, we used only variants in exonic region or variants in ±20 bp flanking those regions. After filtering out variants with a mapping and base-call quality less than 20 and excluding all common variants detectable in more than 1% of the general population (1000 genome, Allele Frequency Community, gnomA, CGI, ExAC, and NHLBI ESP exomes used for comparison), the remaining variants were evaluated for each group using the PolyPhen-2 and SIFT algorithms. Pathogenic, likely pathogenic, and variants of uncertain significance were used to identify genetic variations likely contributing to the phenotype. Genes and pathways that have known involvement in pulmonary hypertension, hypotension, and hemodynamic instability were identified by means of an Ingenuity pathway analysis (Qiagen, Redwood City, CA, USA). Cortisol levels were drawn per the clinical care team’s decision-making pathways where there were symptoms indicating a possible stress response, which could manifest as pulmonary hypertension, hypotension, or hemodynamic instability [3]. As such, these pathways and genes were used as biological filters for the variants due to the physiological response that occurs in the neonatal period after cardiac surgery when adrenal insufficiency may be suspected. 

## 3. Results

Out of 97 neonates, 64 had their cortisol levels drawn in the immediate post-operative period, and, out of these, 16 had NGS panels performed as part of their neonatal care after a baseline microarray. Eight neonates had a cortisol level ≤4.5 mcg/dL, resulting in the CIRCI grouping, and eight had non-CIRCI levels over 4.5 mcg/dL. The cohort demographics, post-operative clinical information, and outcomes of the 16 patients are presented in Table 1 with descriptive statistics by incidence as applicable. All the neonates with CIRCI were treated with dexamethasone after their cortisol levels were drawn, and seven neonates with non-CIRCI were given dexamethasone. 

After removing common variants and low-quality base-calls, 206,409 variants were left for interpretation. Applying the biological filters produced 39 genes with functional connection to pulmonary hypertension and 7 genes linked to hemodynamic instability. No gene mutations were found specific to hypotension. Seven gene mutations with both pulmonary hypertension and hemodynamic instability were found to be present in 75–100% of the neonates with CIRCI (n = 8), with a 0–25% incidence of those corresponding mutations in the neonates in the non-CIRCI group (n = 8), as seen in Table 2. 

The clinically significant importance was evaluated for areas with overlap of these biologic filters and variant interpretations. The CIRCI group had a 100% incidence of heterozygous gene mutation on STX1A with splicing and loss of function. This mutation was not found in the non-CIRCI group. Additional gene mutations were found in the CIRCI group on RAB6A, ABCA3, SIDT2, and LILRB3, with no incidence in the non-CIRCI group. Three additional mutations were found across the CIRCI group in INPPL1 and FAM189A2 (both splicing and missense), with 12–25% of the non-CIRCI group also displaying these mutations. 

## 4. Discussion

This study explored the presence of undiscovered genetic mutations in critically ill neonates who required hydrocortisone supplementation after pediatric cardiac surgery. One heterogeneous genetic mutation had a 100% incidence in the neonates with CIRCI on the STX1A gene with a loss of function. The loss of function on STX1A has potential cardiac intensive care implications given its known pathogenetic processes in clinical and animal models. The additional potential impact from other genes with loss of function or unknown effects remains to be explored for endocrine, pulmonary, and stress pathway implications. 

The descriptive demographics of the cohort were comparable with those of other pediatric cardiac studies evaluating post-operative adrenal insufficiency primarily with term infants over 38 weeks of gestational age, over 80% of whom had single-ventricle physiology, weighting over 2.5 kg at the time of surgery, 75% of whom required cardiopulmonary bypass, and had a mean STAT category of 4 [2]. In our study, 74.2% (n = 72/97) of neonates had a random cortisol level drawn after cardiac surgery as part of their standard clinical care. Of the 97 neonates who had cardiac surgery during the study period, only 16 neonates had both undergone NGS and had their cortisol level drawn as standard clinical care after cardiac surgery. Nearly all the neonates in our study were treated with dexamethasone after a cortisol level had been obtained. 

Many studies have used Scott and Watterberg’s 1995 report on plasma cortisol concentrations for preterm infants as the baseline for neonate cortisol values [10]. We were unable to find normal ranges of cortisol values for term neonates in stress-related periods like secondary adrenal insufficiency after post-cardiac surgery. Based on the existing pediatric adrenocortical insufficiency literature, the values of <3–4.5 mcg/dL as abnormal and >18 mcg/mL for no adrenal insufficiency were used in our group divisions [4,5,6]. All the neonates in our cross-section had a degree of stress-related relative adrenal insufficiency, with values ranging from 0 to 17.8 mcg/dL. No ACTH stimulation tests were conducted in our study. Still, previously, smaller and younger children with more complex congenital heart surgeries had noted abnormalities in their free cortisol responses, which could identify a risk for poorer outcomes [6]. Of note, Wald et al. evaluated total cortisol levels immediately after cardiac surgery and then again 24 h later [6]. 

Due to the retrospective nature of our design, the mean time in days for the cortisol level to be drawn for the CIRCI group was 2.88 days and 8.15 days for the non-CIRCI group. These timings were reflective of when the cardiac care team ordered a cortisol level to be drawn based on the neonates’ clinical hemodynamic status. Relative adrenal insufficiency after cardiac surgery often presents with hemodynamic instability and decreased cardiac output. While not statistically significant, we found that the neonates with CIRCI trended toward a higher rate of pre-operative intubation, post-operative reintubation, reduced transplant-free survival, and longer length of stay in the intensive care unit (Table 1). The CIRCI neonates had a 75% survival rate compared to the 87.5% transplant-free survival rate to discharge due to one cardiac transplant in the non-CIRCI group. In the CIRCI cohort, one patient death was reported at less than 30 days of life, and the other death was at nearly 7 months of age. Wald et al. reported a greater amount of fluid resuscitation and longer length of stay for neonates with a lower total cortisol response to ACTH stimulation testing [6]. We did not evaluate the volume of fluid resuscitation, but 50% of the neonates in our study were on epinephrine infusions at the time the cortisol level was drawn. Along with a higher rate of reintubation (75% CIRCI vs. 37.5% non-CIRCI) and longer overall ventilation days, the CIRCI group had a 37.5% incidence of intubation in the pre-operative period. There was a higher median overall length of stay for the CIRCI group (59.5 days vs. 53.5 days), reflected also in the number of days in the cardiac intensive care unit. These findings show a trend toward higher complications, a longer length of stay, and potentially worse survival rates. 

**Figure 1:**
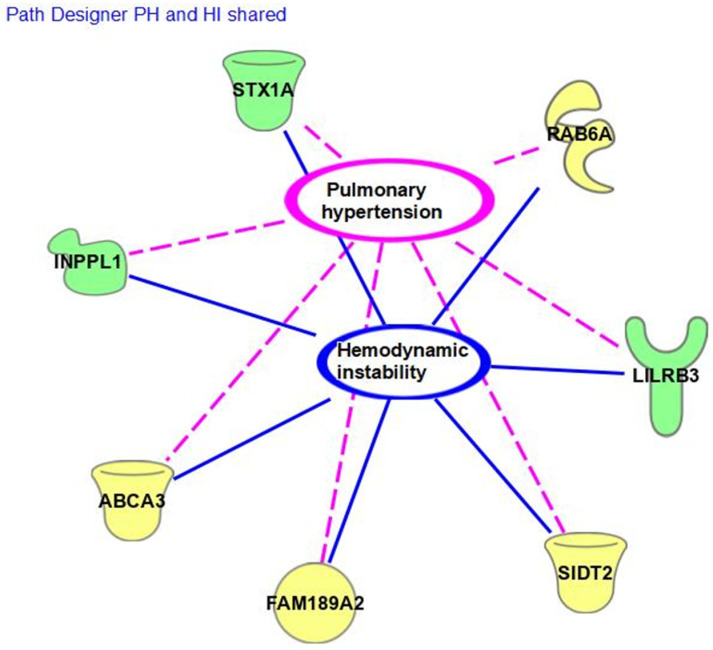
Path designer with key variants for the biologic filters applied. Splicing/loss of function in green. Missense/normal and/or unknown in yellow (Figure made with © 2000-2022 Qiagen software).

The genetic variations outcomes in the CIRCI group, which were not noted in the non-CIRCI group with higher mean levels of cortisol, may lead to further understanding of the variable reactions of some neonates after the stress of cardiac surgery. These gene findings had not previously been reported as a cause for secondary adrenal insufficiency after the stress of cardiac surgery [4]. The genes with identified variants in the CIRCI group vs. the control and their connections to hemodynamic instability and pulmonary hypertension are represented in Figure 1. There are three genes with splicing and impacted loss of function (STX1A, INPPL1, and FAM189A2- c.704-3C>A alteration), two genes with unknown implications, and three with missense and likely normal function. With splicing and loss of function along these pathways, the physiologic implications are more likely to impact a neonate’s response after surgery compared to genes with an unknown change or missense. RAB6A and SIDT2 both had mutations with unknown functions. Missense variation and variant with likely normal function were found for ABCA3, LILRB3, and the second alteration on FAM189A2. A missense mutation means that there are genetic mutations present but that the primary physiological pathways are intact and function normally. 

While these gene variations have yet to be reported in ClinVar, the frequency from the GnomAD global study-wide population is rare at G = 0.00002 (1/45410) [11]. Potential cardiac implications for cardiac intensive care teams were found in the STX1A gene splicing. STX1A is in the SNARE family, which codes the membrane protein syntaxin, responsible for the fusion of synaptic vesicles with the presynaptic plasma membrane. This fusion is accomplished by hydrolyzing ATP to create a conformational change in the protein structure [12]. In animal models, STX1A has been shown to have cardiac implications related to post-operative hemodynamic instability with cardiac dysfunction and excitation–contraction coupling through calcium channel kinetics and K_ATP_ [12]. The systolic dimension and volume were elevated, with a reduced ejection fraction and fractioning shortening in mice models with a loss of STX1A function compared with controls [12]. The molecular changes of a knockout of STX1A function are hypothesized as inhibiting the trafficking of calcium channels, which are localized and voltage-gated. In sarcolemma, there may be an optimization of the excitation–contraction coupling with ryanodine receptors or an insufficiency in calcium channels, causing higher resting cytosolic calcium levels [12]. However, the molecular explanation of the cardiac phenotypic response resulting in the loss of STX1A function still requires further investigation [12]. 

Along with the maintenance of the efficiency of excitation–contraction coupling and myocyte contractility [12], in vivo studies have found that STX1A expression rapidly increased during times of stress, ischemia, and hypoxia and was present in cardiomyocytes [13,14]. Additionally, research on miRNA expression targeted from miR-34a-5P to STX1A evaluated anesthetic sevoflurane through cardio-protection after ischemic cardiomyocyte injury [15]. Additionally, overexpression of miR-34a-5p increased the risk of congenital heart disease in rats [16]. In our study, seven neonates had an echocardiogram performed within 24 h of their cortisol levels being drawn, and 75% (3/4) of the neonates in the CIRCI group were reported to have cardiac dysfunction compared with the 33.3% (1/3) in the non-CIRCI group. The neonates with univentricular and bi-ventricular cardiac anatomies alike had signs of dysfunction, but not all the patients had echocardiograms performed at the same time that related to their cortisol levels, limiting generalizability.

STX1A is also in the critical deletion region for Chromosome 7, found in individuals diagnosed with the contiguous gene deletion disorder or the Williams–Beuren syndrome [17]. Individuals affected by the Williams–Beuren syndrome have been reported to have between 25 and 100 times the risk of cardiovascular-related mortality, adverse outcomes with anesthesia induction, and glucose intolerance compared to non-affected individuals [18]. In our study, no patients were diagnosed with Williams–Beuren syndrome. The phenotypic response of people with this syndrome additionally includes impaired glucose tolerance, which may contribute to the overall response of patients in terms of low cortisol levels [17]. Evaluating this potential impact of the loss of function of STX1A on neonates with CIRCI after cardiac surgery is a critical step in understanding the implications for identifying neonates needing increased corticosteroid support while undergoing times of stress. 

Additionally, INPPL1 and FAM189A2 (also known as ENTREP1) both had splicing and loss of function. INPPL1 encodes for a protein that is an SH2-containing 5’-inositol phosphatase and is involved in regulating insulin function. Numerous publications have related this gene to diabetes and endocrine dysfunction [19]. FAM189A2/ENTREP1 is overexpressed in thyroid and heart tissues [20]. An adult study looking at risk for coronary artery disease with type 1 diabetes found a single nucleotide polymorphism in FAM189A2 [21]. The individual or combined effect of the loss of function on neonates with CIRCI for all three of these genes (STX1A, INPPL1, FAM189A2) is unknown, as there are cardiac and endocrine pathways affected in each. 

The implications of the genetic mutations of RAB6A and SIDT2 are unknown in our population of interest but have been associated with pulmonary, cardiac, and metabolic disorders. RAB6A is a small GTPase involved in vesicular trafficking in endolytic and secretory pathways but has not been reported for clinical significance in ClinVar for a potential pathogenic impact on chromosome 11 [22]. The frequency of this noted mutation in the global study population was G = 0.00008 (2/264690, TOPMED) [22]. Animal studies have also demonstrated that elevated levels of RAB6A RNA and protein were correlated to the development of idiopathic pulmonary fibrosis [23]. With the use of knockout mice to study the gene coding for the RAB6A protein, when exposed to oxidative stress, an increased inflammatory response as well as attenuated alveolar cell death were noted. Across different demographic populations, SIDT2 has been shown to play a role in the development of metabolic syndrome and the eventual progression of coronary artery disease. In a mouse model study conducted by León-Mimila et al., the lipid metabolism pathways involving the SIDT2 gene, which are shared by humans, were shown to be enriched, with a greater gene expression of SIDT2 [24]. 

There may be a limited pathogenic implication of a genetic variance of missense. Our study found that mutations were noted in the neonates with CIRCI in ABCA3, LILRB3, and FAM189A2. ABCA3 dysfunction has also been shown to play a role in the development of pulmonary disorders such as neonatal respiratory distress and interstitial lung disease. As part of the larger ABCA family, the protein coded is responsible for specifically playing a role in the transport regulation of phosphatidylcholine, phosphatidylglycerol, phosphatidylethanolamine, and cholesterol and is critical for pulmonary surfactant homeostasis through the ATP-dependent pathway [25]. LILRB3 codes for one of many leukocyte immunoglobulin-like receptors, one which plays a role in developing our innate immune system. LILRB3 helps to promote the phosphorylation of the molecules STAT and JAK, which are crucial in the development of many biological functions including the induction of cytokine production [26,27]. In our study, FAM189A2 had both one mutation with loss of function for some neonates and some missense mutations. 

The genetic variants’ impact on regulatory pathways in the cardiac, pulmonary, endocrine, and inflammatory response systems may be isolated but may also be impacted in a network, as seen in Figure 1. The compounding effect of these genetic mutations may affect CIRCI phenotype outcomes. The five genes in Table 2 that had no alterations in the non-CIRCI group will have priority in the evaluation of replicability and potential impact among larger samples. Phenotypic responses of short periods of echocardiographic changes after stress, based on genomic variations, will require ex vivo and cardio-physiologic testing. The rare frequency of reporting in a global sample of genomic data for both STX1A and RAB6 mutations points to especially novel areas of interest with a 100% rate in those neonates with CIRCI. 

In addition to identifying those genes predictive for CIRCI, it would be important to refine variants predisposing patients to not developing CIRCI. Future studies identifying these genes may have implications in the volume of steroids administered to a patient pre- and perioperatively as well as in determining whether corticosteroids should be given at all. This information could have a far-reaching impact on the medications administered to patients and on post-operative healing. 

Despite its novel findings, our study has several limitations. First, the sample size was limited and restricted to a single site. Secondly, due to the retrospective and de-identified genetic evaluation in our study, the possibility of offering any interventions was inconceivable, which would otherwise be the goal in clinically managing neonates who have faced these complications. Having access to samples of children from a larger pool of samples as well as multiple sites could help provide insights for generalizability purposes and into potential regional modifiers. 

## 5. Conclusions

Among neonates with CIRCI requiring surgery for congenital heart disease with a post-operative cortisol level equal to or below 4.5 mcg/dL, 100% had a gene heterozygous mutation with loss of function in the STX1A gene from the SNARE family. No neonates in the non-CIRCI group presented this same finding. STX1A has cardiac implications related to post-operative hemodynamic instability with cardiac dysfunction and excitation–contraction coupling through calcium channel kinetics. More studies are needed to investigate these genetic abnormalities with larger multi-site cohorts beyond our single-site evaluation. The long-term potential in this research aligns with the clinical development of a genetic biomarker panel to be performed prior to neonatal cardiac surgery. Identifying predisposition to the development of CIRCI can provide pediatric cardiac healthcare teams with the ability to initiate steroid replacement for adrenal insufficiency to reduce mortality and morbidity risk. 

## Figures and Tables

**Table 1:** Cohort demographics.

n (%) or Median (IQR)	Cohort (n = 16)	CIRCI (n = 8)	Non-CIRCI (n = 8)	*p*-Value
Male gender (%)	13 (81.25)	7 (87.5)	6 (75)	0.500
Race				
White (%) Black (%) Multi-racial (%)	14 (87.5) 1 (6.25) 1 (6.25)	7 (87.5) 1 (12.5) 0	1 (12.5) 0 1 (12. 5)	0.767
Hispanic ethnicity (%)	3 (18.75)	2 (25)	1 (12.5)	0.500
Gestational age (weeks)	39 (1)	39 (0.5)	39 (1)	0.505
Single ventricle cardiac diagnosis (%)	13 (81.25)	7 (87.5)	6 (75)	0.500
Cardiac surgery type				
Central/BTT Shunt (%) COA/Aortic Arch (%) Hybrid (%) Norwood (%) TAPVR/Shunt (%)	8 (50) 2 (12.5) 2 (12.5) 3 (18.75) 1 (6.25)	3 (37.5) 0 2 (25) 2 (25) 1 (12.5)	5 (62.5) 2 (25) 0 1 (12.5) 0	0.825
Age at surgery (days)	10 (7.25)	10 (6.5)	9.5 (5)	0.328
Weight at surgery (kg)	3 (0.66)	3.25 (0.65)	3.42 (0.75)	0.798
Intubated pre-operatively (%)	4 (25)	3 (37.5)	1(4.25)	0.285
STAT category				
1 (%) 2 (%) 3 (%) 4 (%) 5 (%)	1 (6.25) - - 8 (50) 7 (43.75)	- - - 4 (50) 4 (50)	1 (12.5) - - 4 (50) 3 (37.5)	0.48
Cortisol level	5.45 (2.95)	2.15 (3.3)	5.95 (9.98)	<0.0001
Time of day cortisol drawn (00:00)	12:54 (8:33)	15:32 (6:11)	10:32 (10:11)	0.234
Lowest cortisol level drawn (days)	2 (2.55)	2.71 (2.44)	1.11 (2.4)	0.505
Hydrocortisone started after level (%)	15 (93.75)	8 (100)	7 (87.5)	0.500
Length of hydrocortisone (days)	10 (11.25)	10 (7.5)	6 (11)	0.491
ECMO required post-operatively (%)	4 (25)	2 (25)	2 (25)	0.715
Total mechanical ventilation (days)	11 (12.25)	13 (11.5)	9.5 (7)	0.574
Reintubation after initial extubation (%)	9 (56.25)	6 (75)	3 (37.5)	0.442
CBP time (minutes)	123 (29.5)	118.5 (23)	127 (27)	0.394
Open-chest post-operatively	9 (56.25)	5 (62.5)	4 (50)	0.500
Post-operative chest closure (days)	2.5 (8.5)	6 (10.25)	1 (4.25)	0.442
Max VIS first post-operative day	10.5 (12.75)	13 (10.5)	8 (14.75)	0.721
Max Sv02 first post-operative day	48.15 (29.85)	44.3 (18.35)	60.75 (31.2)	0.328
Min right rS02 first post-operative day	36.5 (16)	36.5 (15.25)	35 (24.5)	0.382
Min left rS02 first post-operative day	51.5 (21.25)	48 (16.25)	53 (17)	1.00
Max lactate first post-operative day	3.65 (3.1)	2.8 (1.63)	4.6 (1.63)	0.328
Highest BUN post-operatively	9 (0.25)	9 (0)	9 (1)	0.574
Highest creatinine post-operatively	0.40 (0.24)	0.35 (0.13)	0.43 (0.26)	0.328
Length of CICU stay (days)	26.5 (16)	33 (22.25)	19.5 (12.75)	0.083
Length of neonatal stay (days)	60 (97.25)	59.5 (91.75)	54.5 (90.25)	0.645
Transplant-free survival discharge (%)	13 (81.25)	6 (75)	7 (87.5)	0.500

Note. BTT = Blalock–Taussig–Thomas; CBP = cardiopulmonary bypass; CICU = cardiac intensive care unit; COA = coarctation of the aorta; ECMO = extracorporeal membrane oxygenation; STAT = The Society of Thoracic Surgeons-European Association for Cardio-Thoracic Surgery Score; Sv02 = mixed venous saturation; rS02 = regional oxygenation saturation; TAPVR = total anomalous pulmonary venous repair; and VIS = vasoactive infusion score.

**Table 2:** Genetic mutations in the CIRCI and non-CIRCI groups with pathogenic impact and function.

	Status		Alteration	Impact/Function
	CIRCI	Non-CIRCI		
STX1A	`x x x x x x x x	- - - - - - - -	c.541-8A>C	Splicing/Loss
RAB6A	`- x X X X x X X	- - - - - - - -	c.496-9T>C	Unknown
ABCA3	`x x x x - x x -	- - - - - - - -	c.2695A>C (p.T899P)	Missense/Normal
SIDT2	`- x x x x x - x	- - - - - - - -	c. 1015 + 17T>G	Unknown
LILRB3	`x - x x - x x x	- - - - - - - -	c.940G>Tp.D314Y	Missense/Normal
INPPL1	`x x x x - -x x	- - - - - x - -	c.2329T>Ap.Y777N	Splicing/Loss
FAM189A2	`- x x x x x x x	x - - - - - - -	c.742T>Ap.S248T	Missense/Normal
	`x - x x x x x -	- - x - x - - -	c.704-3C>A	Splicing/Loss

X = homozygous mutation, x = heterozygous mutation, - = no mutations.

## Data Availability

The data presented in this study are available on request from the corresponding author along with a collaborative research plan. The data are not publicly available due to patient confidentiality.
